# A Case of Severe Seronegative Inflammatory Arthritis due to Nivolumab and Review of the Literature

**DOI:** 10.1155/2019/1326734

**Published:** 2019-11-03

**Authors:** Bicky Thapa, Asad Ali, Raunak Nair, Rishik Vashisht, Cassandra Calabrese

**Affiliations:** ^1^Department of Internal Medicine, Cleveland Clinic Fairview Hospital, Cleveland, Ohio, USA; ^2^Neurological Institute, Taussig Center Institute, Cleveland Clinic Foundation, Cleveland, Ohio, USA; ^3^Respiratory Institute, Cleveland Clinic Foundation, Cleveland, Ohio, USA; ^4^Department of Rheumatic & Immunologic Disease, Cleveland Clinic Foundation, Cleveland, Ohio, USA

## Abstract

Immune-checkpoint inhibitors (ICIs) have revolutionized the treatment of cancer, yet therapy is often hampered by immune-related adverse events (irAEs) which range from mild to severe life-threatening events. Musculoskeletal (MSK) irAEs leading to discontinuation of ICIs are uncommon but increasingly recognized. We report a challenging case of severe immune-related seronegative inflammatory arthritis due to nivolumab in a patient with stage IV metastatic adenocarcinoma.

## 1. Introduction

Immune-checkpoint inhibitors (ICIs) have increasingly demonstrated promising outcomes which have led to accelerated approval in many advanced malignancies such as metastatic melanoma, metastatic non-small cell lung cancer, urothelial carcinoma, mismatch repair-deficient solid tumors, head and neck squamous cancers, renal cell carcinoma, and Merkel cell carcinoma [1].

Activated or primed cytotoxic CD8+ T cells along with CD4+ helper T cells identify the antigen present on tumor cells which produces interferon and cytotoxin leading to an antitumor activity [2, 3]. Many tumor cells express program cell death ligand 1 (PD-L1). ICIs target ligand expressed on the surface of T cells [4]. Nivolumab, anti-PD-1 human monoclonal antibody, binds to PD-L1, thereby preventing binding of a ligand to T-cell surface receptor program death 1 (PD-1), subsequently leading to the continued activation of an immune response against tumor cells [4]. Additionally, this mechanism will result in an unrestricted immune response which can lead to immune-related adverse effects (irAEs) affecting various organ systems in the body [5]. The immunologic basis of musculoskeletal irAEs has not been completely elucidated.

We report a rare and challenging case of new-onset seronegative inflammatory arthritis complicated by baker cyst rupture during the course of treatment with nivolumab in a patient with stage IV adenocarcinoma.

## 2. Case Report

A 65-year-old previously healthy male presented with an episode of seizure associated with garbled speech, weakness, and abnormal sensation which on further workup revealed a left frontal brain mass with an unknown etiology which was managed with stereotactic radiosurgery. Whole-body computed tomography (CT) scan showed enlarged lymph nodes in the left supraclavicular area, right hilum, and right aortocaval region. Biopsy of the left supraclavicular lymph node demonstrated poorly differentiated adenocarcinoma with unknown primary; the immune phenotype was not specific and was consistent with metastasis from virtually any visceral organ including lung (pulmonary adenocarcinomas TTF-1 negative 20%). The patient also had elevated CA 19-9, which made it difficult to delineate the primary malignancy site (lung vs. gastrointestinal).

He received six cycles of gemcitabine and carboplatin as first-line therapy; however, restaging scans revealed an increase in lymphadenopathy along with elevated CA 19-9. The patient also received other chemotherapeutic agents (2^nd^ line) but continued to have the progression of the disease. Because of the failure of two lines of chemotherapies, the third line of therapy with nivolumab (3 mg/kg/dose every two weeks) was initiated. The patient reported new onset of mild neuropathy of the hands and feet along with occasional bilateral knee joint pain after two cycles of nivolumab. The joint pain improved on its own; however, the patient continued to have persistent neuropathy. At the time, the differential diagnosis for this patient's neuropathy includes chemotherapy-induced (especially carboplatin), paraneoplastic syndrome, thiamine/B12 deficiency, or nivolumab induced.

The follow-up restaging scan revealed a partial response of the tumor burden after eight cycles of nivolumab. During the course of treatment with nivolumab (after the 10^th^ cycle), the patient also reported fatigue and mild pruritus of hand, which responded to antihistamines.

**Figure 1:**
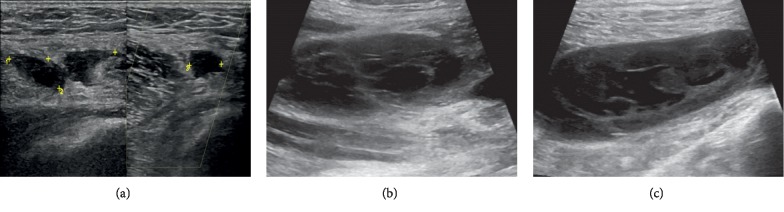
Ultrasound (US) of the left lower extremity: (a) Baker's cyst. (b) Follow-up US showing the increased size of baker's cyst. (c) Image depicting ruptured Baker's cyst.

**Figure 2:**
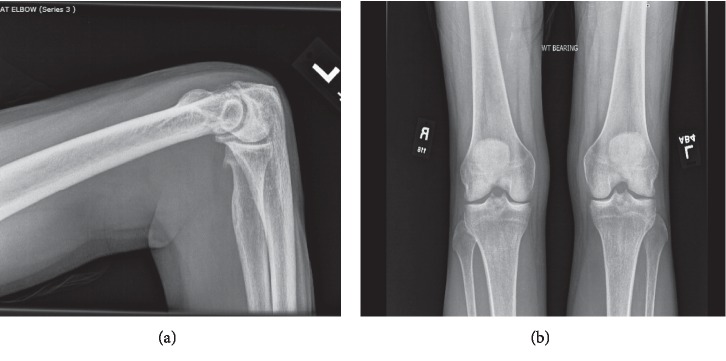
X-ray of the left elbow joint and bilateral knee joints. (a) Moderate osteophytosis at the humeroulnar articulation and involving the radial head with mild joint space narrowing and no joint effusion. (b) Anterior-posterior weight-bearing view; no acute fracture/dislocation, and joint spaces grossly preserved.

Subsequently, after the 11^th^ cycle of nivolumab, the clinical course was complicated by joint pain involving knees, elbows, and great toes associated with joint stiffness, swelling, and muscle weakness. Physical examination was significant for left leg swelling along with calf tenderness, and muscle strength was noted to be 5/5 in bilateral knee and ankle joints. Routine blood workup including the liver function test and creatine phosphokinase (CPK) was within the normal range. Ultrasound (US) of the left lower extremity demonstrated a Baker's cyst measuring 3.7 × 0.9 × 1.1 cm (Figure 1(a)) which increased to 8.0 × 6.5 × 2.3 cm (Figure 1(b)) on repeat US four days later. Follow-up US after two weeks revealed cyst rupture with hematoma (Figure 1(c)). At this point, the patient was experiencing severe left knee pain that affected his mobility and quality of life. The patient was also evaluated by orthopedics for the left knee pain and calf swelling, as well as elbow pain and swelling. X-rays revealed unremarkable left knee joint and findings consistent with osteoarthritis of the left elbow (Figure 2).

Based on clinical evaluation and worsening symptoms, the patient was started on prednisone 20 mg twice daily for severe immune-related arthritis with improvement in symptoms. However, on a tapering dose of steroids, the patient had worsening arthritis and neuropathy as well.

The patient reported improvement in arthritis symptoms with an initial dose of prednisone (20 mg twice daily dose) but had an intermittent flare-up of arthritis with a change in weather and exertion.

Rheumatology was consulted and started on weekly oral methotrexate 10 mg (MTX) along with prednisone 10 mg daily. The RAPID-3 score (routine assessment of patient index data 3) [6] was 4.4 on the initial evaluation, indicating low disease severity. Laboratory evaluation revealed a positive antinuclear antibody (ANA) with the titer of 1 : 160 (Table 1); the remainder of autoimmune serology was negative (Table 1).

The patient's joint symptoms were well controlled on MTX and prednisone; however, arthritis worsened upon tapering of prednisone, so MTX dose was increased from 10 to 20 mg weekly. After six months of treatment, his RAPID-3 score was 3.9, prednisone was ultimately tapered off, and hydroxychloroquine and sulfasalazine were added. Five months later, his RAPID-3 was 1.3, and he was off DMARDs and did not experience recurrence of rheumatic irAE.

**Figure 3:**
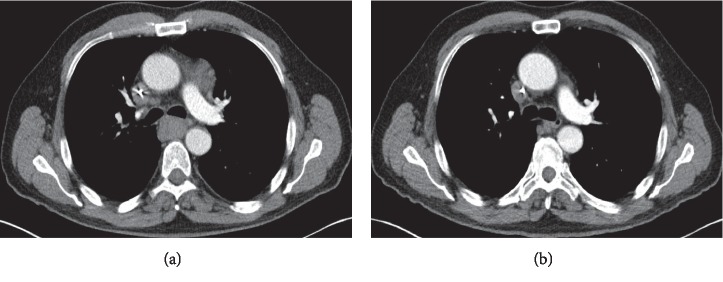
Computed tomography (CT) scan of the chest with contrast before and after nivolumab. (a) CT chest before initiation of nivolumab; an axial image depicting significant mediastinal and right hilar lymphadenopathy. (b) CT chest after 12 cycles of nivolumab; no hilar or mediastinal lymphadenopathy identified.

**Figure 4:**
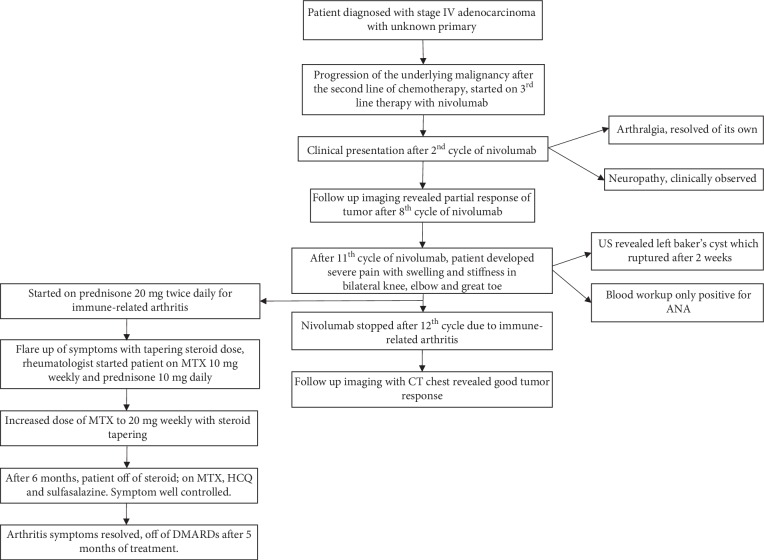
Flowchart illustrating the complete clinical course of the patient. ANA: antinuclear antibody, US: ultrasound, MTX: methotrexate, HCQ: hydroxychloroquine, DMARDs: disease-modifying antirheumatic drugs.

Although the patient's tumor responded to nivolumab (Figure 3), it was stopped after 12 cycles due to MSK irAE. Unfortunately, the patient had severe inflammatory arthritis which worsened the quality of life due to nivolumab; the shared decision was made not to reintroduce nivolumab (Figure 4).

In summary, our patient had initial irAEs in the form of grade 1 neuropathy and grade 1 inflammatory arthritis which resolved on its own; but subsequently, during the treatment course with anti-PD1, the patient developed fatigue and grade 1 pruritus which improved with an antihistamine. Eventually, our patient had grade 2 inflammatory arthritis based on the recommendations from the society for the immunotherapy of cancer toxicity management working group [7], which was managed with glucocorticoids, DMARDs, and discontinuation of ICI.

## 3. Discussion

A better understanding of irAEs is crucial to ensure the safety and quality of care for cancer patients. The spectrum of irAEs is quite diverse, and while they have been described in essentially every organ system, the most common irAEs are dermatologic, gastrointestinal, and endocrine toxicities such as hypothyroidism and hypophysitis [5, 8, 9]. Less common but life-threatening toxicities include myocarditis, amongst others [10–12].

Arthralgia and myalgia are the most frequent musculoskeletal (MSK) irAEs and have been reported up to 40% in clinical trials [13–15]. However, severe immune-mediated arthritis is rare and has been described in the literature in the form of case reports and case series [13, 14, 16–21].

MSK irAEs have been manifested in varied clinical forms such as arthralgia, myalgia, myositis, fasciitis, tenosynovitis, mild to severe arthritis, psoriatic arthritis, and polymyalgia rheumatica (PMR) (Table 2). Early recognition of rheumatological irAEs and management by rheumatologists play a crucial role in improving outcomes in patients receiving ICI and the continuation of ICI for the treatment of underlying malignancy [15].

As per the literature review, MSK irAEs have heterogeneous phenotypical presentation, mostly symmetrical polyarthritis associated with synovitis. Clinical manifestation varies from large joint oligoarthritic to polyarthritis involving small joints, sometimes accompanied by reactive symptoms such as conjunctivitis and urethritis [13]. Furthermore, some of the most recent case series also reported myositis and fasciitis due to ICI, broadening the clinical presentations of MSK irAEs [33, 34].

While popliteal (Baker's) cyst has not been specifically described in the literature as being a manifestation of rheumatic irAEs, it is well known that rates of popliteal cysts are higher in the patients with inflammatory arthritis, including rheumatoid arthritis [35].

Rheumatic auto-antibodies such as rheumatoid factor (RF) and anti-CCP antibody are generally negative [33, 34]; however, there are case series and case reports of seropositive rheumatoid arthritis [26, 27, 30, 32, 33]. Our patient had a low-positive ANA, which was unlikely to be of any clinical significance, as this is a nonspecific test, and his clinical picture was not consistent with an autoimmune connective tissue disease such as systemic lupus erythematosus and the more specific auto-antibodies were negative. To date, there has been no association found between positive ANA and the development of rheumatic irAEs.

It has been observed from most of the case series and case reports, MSK irAEs are more prevalent in the patient population treated with an anti-PD1 single agent than in combination with anti-CTLA-4; only two patient has been found to have inflammatory arthritis after single-agent anti-CTLA-4 [31, 32]. There are rare irAE reports of psoriatic arthritis which responded well to either steroid alone or in combination with synthetic DMARDs [22, 23, 27, 32].

Patients also demonstrated a variable clinical course with unpredictable onset and difference in response to different therapeutic agents such as nonsteroidal anti-inflammatory drugs (NSAIDs), steroid, intraarticular steroid, synthetic, or biologic DMARDs. Most of the patients had a good response with a low to moderate dose of steroid alone, and DMARDs were used to taper off the steroid for better control of the symptoms [25–29, 31, 33, 34]. Moreover, refractory MSK irAEs required biological DMARDs and, in some cases, anti-IL-6 or tumor necrosis factor inhibitor alfa (TNF) with improvement in symptoms [13, 14, 24]. Patients with PMR responded well to steroids with rapid improvement but required TNF alfa inhibitors in one of the cases [14].

Our patient's tumor responded to nivolumab (Figure 3); however, ICI was discontinued due to immune-related arthritis. Most of the cases in th epublished literature did not require discontinuation of the ICIs [26, 32, 33, 34]; in fact, it has been proposed that MSK irAEs are associated with better tumor outcomes from ICI. Kostine et al. reported the incidence of rheumatic irAEs of 6.6% [32] andthe authors also observed higher tumor response in a patient who had rheumatic irAEs and emphasized the continuation of ICIs rather than discontinuation for management of MSK irAEs.

Rheumatic irAEs are different in that they may be chronic in up to one-third of the patients after cessation requiring long-term management [4, 15, 36]. Corticosteroid is the mainstay of treatment for immune-related arthritis; however, in refractory cases, it is recommended to consider either synthetic or biologic DMARDs [4, 37]. Besides, it is also recommended to stop ICIs in severe cases of inflammatory arthritis and permanently discontinue ICIs in grade 4 toxicities [4, 15, 37]. In our case, the patient had refractory severe immune-mediated inflammatory arthritis requiring to stop ICI, and he achieved remission on the initiation of DMARD during the course of the treatment. Interestingly, the clinical course was further complicated by ruptured Baker's cyst. Although the underlying mechanism for the rupture of Baker's cyst in our patient is unknown; however, nivolumab could be the possible reason based on clinical presentations and temporal relationships. Nevertheless, seronegative rheumatological workup and nonspecific positivity of ANA is consistent with previously published literature.

In conclusion, musculoskeletal irAEs are relatively common. Most patients on ICIs experience mild symptoms which often resolve with glucocorticoids alone. However, immune-related inflammatory arthritis requiring conventional or biologic DMARD therapy remains a clinical challenge. It is of utmost importance to recognize these cases and manage in collaboration with a rheumatologist to improve the clinical outcome and quality of life.

## Figures and Tables

**Table 1:** Immunologic workup.

Immunology/serology	Reference range	Result
ANA	Negative	Positive
ANA titer	Negative	1 : 160
ANA pattern	Unknown	Nucleolar
DNA antibody	<30 IU/mL	22
RNP antibody	<1.0	<0.2
Ribosomal RNP	<1.0	<0.2
SSB antibody	<1.0	<0.2
Sm antibody	<1.0	<0.2
SSA antibody	<1.0	<0.2
Scleroderma Ab, IgG	<1.0	<0.2
Jo 1 antibody	<1.0	<0.2
Chromatin antibody	<1.0	<0.2
Centromere Ab	<1.0	<0.2
Rheumatoid factor	<16 IU/mL	<10
CCP antibody, IgG	<20 units	<15

ANA: antinuclear antibody; DNA: deoxyribonucleic acid; RNP: ribonucleoprotein; SSB: anti-Sjögren's syndrome type B; SSA: anti-Sjögren's syndrome type B; Ab: antibody; CCP: cyclic citrullinated peptide; IU: international unit.

**Table 2:** Summary of published case series or case report on rheumatologic/musculoskeletal immune-related adverse effects (irAEs).

Case series/report(s)	Number of patients	Immune-checkpoint inhibitor	Primary malignancy	Pattern	Joint involvement	Symmetrical/Asymmetrical	Axial/appendicular	Clinical diagnosis	Serology	Therapy	Steroid dose (for arthritis patients only)	Response	ICI status
Chan et al. [17]	2	Anti-PD1: 2	Melanoma: 2	Seronegative arthritis: 2	Polyarthritis: 2	NA	None	Inflammatory arthritis	RF: −ve, anti-CCP: −ve, ANA: −ve	Opioid analgesics: 1, pamidronate, NSAIDs and DMARDs (sulfasalazine, HCQ): 1	Steroid not used	Improvement: 2	Continued: 1, held: 1
Law-Ping-Man et al. [22]	1	Anti-PD1	NSCLC	Psoriatic arthritis	Oligoarthritis	Asymmetrical	None	Inflammatory arthritis	RF: −ve, anti-CCP: −ve, ANA: −ve	Steroid, DMARDs (MTX)	Prednisone (15 mg/day)	Improvement	Continued
Ruiz-Bañobre et al. [23]	1	Anti-PD1	NSCLC	Psoriatic arthritis	Polyarthritis	NA	NR	Inflammatory arthritis	RF: −ve, anti-CCP: −ve, ANA: −ve	IA steroid, steroid, NSAIDs, DMARDS (MTX, sulfasalazine)	Methylprednisone (4 mg/day)	Improvement	Continued
Kim et al. [24]	3	Anti-PD1: 1, anti-PD1 and anti-CTLA-4: 2	Melanoma: 3	Seronegative arthritis: 3	Polyarthritis: 3	Symmetrical: 3	None	Arthritis	RF: −ve, anti-CCP Ab: −ve, ANA: 1	Prednisone and anti-IL-6 Ab: 2, anti-IL-6 Ab (tocilizumab): 1	Prednisone (40 mg/day)	Improvement: 3	NR
Kuswanto et al. [19]	4	Anti-PD1: 4	RCC: 4	Seronegative arthritis: 3, PMR: 1	Polyarthritis: 3	Symmetrical: 2	Pelvic girdle: 1	Inflammatory arthritis	RF: NA, anti-CCP Ab: NA, ANA: NA	Steroid: 1, steroid and IA steroid: 1, steroid and DMARDs (MTX): 1, infliximab: 1	Prednisone (50–20 mg/day)	Improvement: 4	Held: 3
Cappelli et al. [25]	9	Anti-PD1: 2, anti-PD1 and anti-CTLA-4: 7	RCC: 1, small cell lung cancer: 1, melanoma: 3, NSCLC: 4	Seronegative arthritis: 9	Polyarthritis: 8, oligoarthritis: 1	NR	Back pain: 1	Inflammatory arthritis	RF: −ve, anti-CCP Ab: −ve, ANA: 1	Prednisone: 1, dexamethasone: 1, NSAIDs and IA steroid: 1, prednisone and TNF alfa inhibitor: 2, prednisone, DMARDs (MTX, infliximab, etanercept) and IA steroid: 2, prednisone and IA steroid: 2	Prednisone (40–10 mg/day), 120 mg for 1 patient	Improvement: 7, partial response: 1, NR: 1	NR
Calabrese et al. [20]	10	Anti-PD1: 1, anti-PD1 and anti-CTLA-4: 6	NSCLC: 1, RCC: 2, melanoma: 4	Seronegative arthritis: 7, PMR: 3	Polyarthritis: 7	Symmetrical: 6, asymmetrical: 1	None	Arthritis	RF: 1, anti-CCP Ab: −ve, ANA: 1	Prednisone: 6, prednisone and DMARDs (HCQ, MTX, infliximab, etanercept, adalimumab): 4	Prednisone (40–15 mg/day)	Significant improvement: 4, moderate improvement: 4, minimal improvement: 2	Held: 4, continued: 3
Belkhir et al. [26]	10	Anti-PD1/PD-L1: 9, anti-PD1 and anti-CTLA-4: 1	Melanoma: 3, lung adenocarcinoma: 2, endometrial adenocarcinoma: 1, squamous cell carcinoma of the vagina: 1, mesothelioma: 1, colon adenocarcinoma: 1, gastric adenocarcinoma: 1	RA: 6, PMR: 4	NR	NR	NR	Inflammatory arthritis	RF: 4, anti-CCP Ab: 6, ANA: 1	Steroid: 7, NSAIDs: 1, NSAIDs and DMARDs (MTX, HCQ): 2	Prednisone (20–10 mg/day) 60 mg for 1 patient	Improvement: 10	Continued: 9
Le Burel et al. [27]	17	Anti-PD1/PD-L1: 15, anti-PD1 and anti-CTLA-4: 2	Melanoma: 3, RCC: 2, lung adenocarcinoma: 3, pleural mesothelioma: 1, endometrioid carcinoma: 1, colon adenocarcinoma: 1, vaginal squamous cell carcinoma: 1, lung squamous carcinoma: 1, urothelial bladder cancer: 1, glioblastoma: 1, renal epithelioid angiomyolipoma: 1, gastric adenocarcinoma: 1	RA: 3, PMR: 4, psoriatic arthritis: 3, seronegative arthritis: 7	Polyarthritis: 13	Symmetrical: 11, asymmetrical: 1	NR	Inflammatory arthritis	RF: 3, anti-CCP Ab: 3, ANA: 1	Steroids: 14, steroid and DMARDs (MTX): 2	NA	Improvement: 9, resolution: 7, stable: 1	NA
Le Bakhaya et al. [28]	26	Anti-PD1: 23, anti-PD1 and anti-CTLA-4: 3	Melanoma: 25, Merkel cell carcinoma: 1	Arthritis: 10, activated osteoarthritis: 5, arthralgia (not specified): 11	Polyarthritis: 7, oligoarthritis: 19	Symmetrical: 16	NR	Arthralgia	RF: 2, anti-CCP ab: 1, ANA: 1	NSAIDs: 19, steroid: 5, DMARDs (sulfasalazine, HCQ): 2	Prednisone (5–10 mg/day)	Complete response: 4, partial response: 15, stable disease: 5	Held: 2
Ngo et al. [29]	1	Anti-PD1	Melanoma	Seronegative arthritis	Polyarthritis	Symmetrical	None	Inflammatory arthritis	RF: −ve, anti-CCP: −ve, ANA: −ve	Steroid	Prednisone (40 mg/day)	Improvement	Continued
Haikal et al. [21]	1	Anti-PD1	Melanoma	Seronegative arthritis	Polyarthritis	Symmetrical	None	Arthritis	RF: −ve, anti-CCP: −ve	Steroid and DMARDs (HCQ)	Low-dose steroid	Improvement	NR
Inamo et al. [18]	3	Anti-PD1: 3	Lung adenocarcinoma: 1, lung squamous cell carcinoma: 1, ovarian cancer: 1	Seronegative arthritis: 3	Polyarthritis: 2, oligoarthritis: 1	Symmetrical: 3	None	Inflammatory arthritis	RF: −ve, Anti-CCP Ab: −ve, ANA: −ve	NSAIDs: 1, opioid: 1, NSAIDs and prednisone: 1	Prednisone (20 mg/day) for 1 patient only	Improvement: 3	NR
Smith and Bass [30]	10	Anti- PD1: 3, anti-PD-1 and anti-CTLA-4: 7	Melanoma: 4, lung adenocarcinoma: 2, RCC: 1, Merkel: 1, anal cancer: 1, cervical cancer: 1	RA: 3, PMR: 1, others: 6	Polyarthritis: 4, oligoarthritis: 4, tenosynovitis: 2	NA	NA	Inflammatory arthritis	RF: NA, anti- CCP Ab: 2, ANA: 6	Steroid: 6, steroid and DMARDs (HCQ, sulfasalazine, MTX, mycophenolate mofetil): 4	Prednisone (20–10 mg/day)	Improvement: 4, resolution: 6	Held: 1
Lidar et al. [31]	12	Anti-PD1: 8, anti-CTLA 4: 1, anti-PD1 and anti-CTLA-4: 3	Melanoma: 10, sinonasal carcinoma: 1, endometrial carcinoma: 1	Seronegative arthritis: 12	Polyarthritis: 10, oligoarthritis: 1, monoarthritis: 1	NR	None	Inflammatory arthritis	RF: −ve, anti-CCP Ab: none, ANA: −ve	NSAIDs, steroid: 5, steroid and MTX: 2, NSAIDs, steroid, MTX: 5	Prednisone (>20 mg/day)	Improvement: 11, unknown: 1	Held: 3, continued: 3, off therapy: 6
Kostine et al. [32]	35	Anti-PD1/PD-L1: 33, anti-CTLA-4: 1, anti-PD-L1 and anti-CTLA- 4: 1	Melanoma: 16, lung cancer: 12, RCC: 6, Merkel cell carcinoma: 1	RA: 7, PMR: 11, psoriatic arthritis: 2, others: 15	Mostly polyarthritis: exact no. not reported	Mostly symmetrical	Back pain: 10	Inflammatory and noninflammatory arthritis	RF: −ve, anti-CCP Ab: 1, ANA: 4	Steroid: 18, NSAIDs: 5, steroid and DMARDs (MTX): 2, intraarticular steroid: 1	Prednisone (mean dose 15 mg)	Improvement: 35	Continued: 34
Leipe et al. [33]	14	Anti-PD1: 10, anti-PD1 and anti-CTLA4: 4	Melanoma: 10, NSCLC: 5	Arthritis: 14, PMR: 5, Sicca syndrome: 2, myositis: 1	Polyarthritis: 2, oligoarthritis: 5, monoarthritis: 7	NR	NA	New-onset arthralgia and arthritis	RF: 5, anti-CCP: 1, ANA: 9	Steroid: 3, steroid and DMRDs (MTX): 6, NSAIDs: 2, IA steroid: 8	NR	Improvement: 13	Continued: 14
Narváez et al. [34]	11	Anti-PD1/PD-L1: 10, anti-PD1 and anti-CTLA4: 1	Lymphoma: 2, lung cancer: 4, RCC: 1, epithelioid mesothelioma: 1, pancreatic neuroendocrine cancer: 1, melanoma: 1, urothelial carcinoma: 1	Seronegative arthritis: 5, PMR: 1, Sicca syndrome: 2, inflammatory myositis with fasciitis: 2, paraneoplastic acral vascular syndrome: 1	Polyarthritis: 4, oligoarthritis: 1	Mostly symmetrical	None	Arthritis	RF: −ve, anti-CCP: −ve, ANA: −ve	Steroid: 4, NSAIDs: 1, steroid and DMARDs (HCQ): 3	Prednisone (cobra light schedule): 2 patients, prednisone 60 mg daily for 1 patient and methylprednisone 20 mg daily for 1 patient	Improvement: 10	Continued: 6

## Abbreviations

PD1: Programmed cell death protein 1
CTLA-4: Cytotoxic T-lymphocyte-associated protein 4
NSCLC: Non-small cell lung cancer
RCC: Renal cell carcinoma
RA: Rheumatoid arthritis
PMR: Polymyalgia rheumatica
RF: Rheumatoid factor
Anti-CCP Ab: Anti-cyclic citrullinated peptide antibody
ANA: Antinuclear antibodies
−ve: Negative
NA: Not available
NR: Not reported
DMARDs: Disease-modifying antirheumatic drugs
MTX: Methotrexate
HCQ: Hydroxychloroquine
NSAIDs: Nonsteroidal anti-inflammatory drug
IA steroid: Intra-articular steroid.
